# Know Your Nose: A Narrative Review of the Developmental and Functional Impact and Importance of the Nose, Nasal Breathing and Techniques on Health and Emotional Wellbeing

**DOI:** 10.3390/bs16030467

**Published:** 2026-03-21

**Authors:** Alireza Amirsadri, Hooman Sedighi

**Affiliations:** 1 Department of Psychiatry and Behavioral Neurosciences, School of Medicine, Wayne State University, Detroit, MI 48201, USA; 2 Physical Medicine Consultants, Dallas, TX 75235, USA

**Keywords:** nasal breathing, autonomic nervous system, nitric oxide, traditional breathing practices, psychological wellbeing

## Abstract

This narrative review synthesizes research demonstrating the multi-domain import of nasal breathing across developmental, physiological, immunological, and neuropsychological domains, with the aim of communicating its potential clinical relevance and motivating future empirical investigation. We broadly address developmental and evolutionary foundations and the pathways through which nasal breathing influences health, functioning, and subjective experience. Across these areas, evidence implicates nasal breathing in immune defense, autonomic and emotion regulation, limbic entrainment, and aspects of consciousness. Notably, many contemplative traditions—including yogic pranayama, Sufi, and Buddhist practices—have long emphasized nasal breathing for its physical and spiritual benefits, and contemporary evidence increasingly buttresses components of these traditional beliefs, with growing convergence between contemporary scientific findings and longstanding traditional observation. More broadly, the epistemic basis of the evidence supporting nasal breathing’s effects reviewed here ranges considerably, from well-controlled experimental and mechanistic work to preliminary and small-sample studies whose clinical translation remains tenuous, and specific therapeutic inferences should be made cautiously. Nonetheless, nasal breathing represents an underappreciated, low-cost, and accessible adjunctive approach with genuine clinical potential. Realizing that potential will require controlled trials attending to parameter specificity—e.g., respiratory phase, laterality, and rate—and designs that isolate nasal breathing from other aspects of contemplative practices across well-defined populations and outcomes.

## 1. Introduction

Scientific advances in recent decades have shed light on the significance of nasal breathing and the role of the nose, revealing its involvement across a spectrum of domains (Heck et al., 2019; Trabalon & Schaal, 2012; Zelano et al., 2016). Deep breathing techniques, especially nasal breathing, have garnered attention for their putative impact on stress reduction and relaxation (Zaccaro et al., 2018), and have become almost mainstream in medical communities under the umbrella of alternative and complementary medicine, as well as in mind–body practices in popular culture (Jerath et al., 2015; Kang, 2010). Notably, many of these practices have been preserved and transmitted across centuries through contemplative traditions.

In the breathing practices of Eastern cultures such as Buddhism, Hinduism (McAllister, 2020), Kabbalah in Judaism (Zemmelman, 2017), and Tamarkoz in Islamic Sufism (Bahadorani et al., 2021) nasal breathing has been integral, where it is used with the aim of attaining higher levels of spiritual consciousness. These phenomenological experiences, as described by practitioners, may correspond to measurable physiological changes such as altered Default Mode Network (DMN) activity and interoceptive awareness, and increased parasympathetic tone (Bahadorani et al., 2021; Zaccaro et al., 2022).

In the following text, we provide an interdisciplinary discussion of the wide-reaching, discipline-spanning significance that nasal breathing holds. These topics include evolutionary accounts of the structure and function of the nose and sinuses and their relevance to nasal breathing; nasal breathing’s associations with morphological and autonomic nervous system development; its immunological functions; its role in olfaction; its neurological and psychological effects; its relationship to consciousness; and its place in ancient spiritual practices.

Given this breadth and our aim of communicating the importance of nasal breathing to clinicians, we employed a classical narrative review approach to synthesize the literature on the neurophysiological mechanisms and health implications of nasal breathing. In doing so, we address three broad questions: (1) What are the developmental and evolutionary foundations of nasal breathing? (2) What are the key physiological mechanisms through which nasal breathing influences health? (3) What is the evidence that specific nasal breathing practices contribute to emotional wellbeing and psychological outcomes? Unless otherwise specified, ‘nasal breathing’ refers to the complete respiratory cycle, encompassing both inhalation and exhalation through the nose.

## 2. Methods

### 2.1. Design

Narrative reviews are appropriate when integrating diverse bodies of evidence across multiple disciplines to provide a descriptive analysis and scope of a topic (Ferrari, 2015; Greenhalgh et al., 2018). We adopted a narrative review approach, which prioritizes conceptual integration and thematic synthesis over exhaustive retrieval or formal quality appraisal (Greenhalgh et al., 2018). This approach guided both selection and synthesis in which sources were identified and prioritized based on their relevance to the thematic domains rather than through standardized extraction protocols. The findings were organized thematically to highlight connections across disciplines.

### 2.2. Eligibility Criteria

Sources were included if they: (a) addressed physiological, neurological, or psychological influences of nasal breathing; (b) examined developmental aspects of nasal breathing; (c) investigated nasal breathing practices such as pranayama, Tamarkoz, coherent breathing, or other controlled nasal respiration techniques; (d) provided evidence on nasal nitric oxide production and its physiological effects; (e) examined comparative aspects of nasal versus oral breathing on health and wellbeing.

We included peer-reviewed empirical (experimental, observational, case reports, clinical) and review articles, and historical/religious texts relevant to the cultural context of nasal breathing practices. No restrictions were placed on study populations, model systems, publication language, or publication date.

### 2.3. Search Strategy

Literature searches were conducted in PubMed and PubMed Central (PMC). Domain-specific terms were anchored to nasal breathing and/or nasal respiration and included combinations of the following: nasal structure, evolution, nasal morphology, paranasal sinus function, craniofacial development, olfactory, nitric oxide, mucosal immunity, antimicrobial, vagus nerve, parasympathetic, autonomic nervous system, breastfeeding respiration, infant breathing, pranayama, Tamarkoz, coherent breathing, unilateral nostril breathing, respiratory sinus arrhythmia, limbic system, amygdala, consciousness, default mode network, interoception, anxiety, stress reduction, emotion regulation, and wellbeing. Reference lists of retrieved articles were reviewed to identify additional relevant sources. The search was not intended to be exhaustive of any single domain; rather, the aim was to synthesize key findings across multiple areas to communicate the importance of nasal breathing to a clinical audience. The first author conducted the searches. The final search was conducted in December 2025.

Searches were conducted using ad hoc combinations of the above terms, guided by emerging themes and cross-references rather than a pre-specified Boolean search protocol. Table 1 presents our query strategy organized by thematic domain. This approach is consistent with the exploratory nature of narrative reviews, in which the search process is typically recursive and guided by the reviewer’s evolving understanding of the literature (Ferrari, 2015).

### 2.4. Study Selection and Data Extraction

The titles and abstracts were screened for relevance to the above topics. The full text of potentially relevant articles was then judged against the eligibility criteria described above. No formal extraction form was used. Instead, the information was organized thematically according to the article’s topic. Approximately 70 sources were included.

### 2.5. Quality Appraisal

Articles were appraised for relevance to our review’s objective rather than formally assessed for methodological quality. However, excepting for the religious texts, all articles included were sourced from peer-reviewed sources, ensuring a baseline standard of scientific rigor. Table 2 summarizes the key findings, designs, and evidence characterization across domains; the following sections discuss each in greater detail.

## 3. Narrative Synthesis

### 3.1. Developmental Foundations: Nasal Breathing in Infancy

To provide a more concise analysis, we first review some relevant developmental information. The significance of nasal breathing begins in infancy, though as subsequent sections will demonstrate, extends throughout the lifespan. While genetic and environmental factors that play a major role in the development of facial bones and structures such as the maxilla, mandible, teeth organization and alignment (Andriani et al., 2021), nasal breathing during the infant’s breastfeeding is thought to contributes to the development and proper growth of sinuses and a well-balanced facial structure (Cudziło et al., 2018). Without meaningful nasal breathing, the infant cannot latch onto the mother’s breast correctly (Poskitt, 1988). This act results in creating a vacuum or negative pressure in the mouth, with proper utilization of mouth, tongue, and pharyngeal muscles with their repetitive and coordinated movements, crucial for infant feeding and the development of facial and oral formations and functions (Moral et al., 2010). In addition, Gipson et al. (2019) have demonstrated the importance of nasal breathing and breastfeeding in the reduction in the rate of obstructive sleep apnea in an individual’s life.

In the minutes to hours following birth, after the intense physical demands of labor and delivery, the infant’s most significant calming and stress-reducing experience begins with breastfeeding. The multisensory nature of breastfeeding, encompassing touch, warmth, familiar gentle pressure, maternal scent and voice, along with the deeper nasal breathing required for feeding, creates a profound calming experience (Feldman & Eidelman, 2003).

Nasal breathing during breastfeeding stimulates the sensory mechanoreceptors of vagus and trigeminal nerves in the infant’s nasal cavity (Heck et al., 2017). As a result of the rhythmic and repeated act of nasal breathing during suckling, parasympathetic tone is enhanced, contributing to the maturation and durability of autonomic regulatory functions, including heart rate variability (HRV), digestive function, and emotion regulation (Feldman & Eidelman, 2007).

Due to its ancient role in survival, the olfactory bulb, through the olfactory tract, bypasses the relaying and filtering functions of the thalamus and directly connects to the amygdala, enabling rapid survival responses and the formation of powerful emotional memories. Olfactory modification of amygdala activity during breastfeeding reduces stress neurotransmitters and hormones while beneficial hormones and peptides such as oxytocin, endorphins, serotonin, and GABA are released, promoting calmness, bonding, and safety in the infant (Uvnäs-Moberg, 1998). With repetition of these processes, the infant’s brain establishes strong associative memories faster than at any other time in life, creating permanent and stable neural pathways due to the high synaptic plasticity of infancy (Feldman & Eidelman, 2003).

### 3.2. Evolutionary and Structural Adaptations of the Nose

From an evolutionary standpoint, the structure and shape of the human nose putatively evolved to enhance olfaction, respiration, safe airway passage, and lung safety, all aimed at maximizing survival (Roseman & Auerbach, 2015). The bony structures of the nasal turbinates have evolved to increase the nasal mucosal surface area and to create airflow turbulence, warming up and humidifying the entering air. Similarly, external structures such as the nostril size and shape may vary among human populations for this purpose. For instance, in colder areas, the nostrils may become narrower to control the flow of cold air and to allow more time for humidification and warming (Kelly et al., 2020).

Nasal structures, such as the curved shape of the nose, nostrils, and the coarse hairs in the nasal vestibule, also serve as a first line of defense against pathogens and airborne particles (Zaidi et al., 2017). Paranasal sinuses (sphenoid, ethmoid, frontal, and maxillary) not only reduce the weight of the skull, produce resonance for the voice, and protect the brain from trauma but also contribute significantly to more efficient and healthy breathing (Jankowski et al., 2016). Sinus mucosa trap small particles such as pollen and dust, preventing their entry into the lungs, and contain immune cells and antibodies to guard against potential damage from pathogenic agents such as microorganisms.

### 3.3. Nitric Oxide: Production, Function, and Immune Defense

Through nitric oxide (NO) production, the sinuses play a notable role in immune defense, pulmonary function, and vascular regulation. NO is a gasotransmitter produced by a family of enzymes called nitric oxide synthase (NOS) from the amino acid arginine (Kourosh-Arami et al., 2020). NOS is found in blood vessel endothelial cells, neurons, and cells participating in inflammatory processes. NO serves diverse physiological functions: it acts as a neuromodulator^1^, serves as an anti-inflammatory agent, and plays a key role in the human immune response and destruction of biological microorganisms (Bauer & Sotníková, 2010). Through its vasodilatory effect, it regulates blood pressure and contributes to the maintenance of cardiovascular health (Infante et al., 2021).

After production in paranasal sinuses, NO is directed to the lungs. The degree of NO concentration depends on the continuity and rate of airflow. In an intriguing clinical investigation, researchers compared pulmonary arterial oxygenation between two groups of intubated patients: those deprived of their own nasal NO and those who artificially received their own NO through their ventilator’s inhalation limb (J. O. N. Lundberg et al., 1996). In all study subjects, long-term intubated patients who artificially received their own low-dose NO derived from their nose, experienced an 18% increase in their pulmonary artery oxygenation. Moreover, in another arm of the same study, transcutaneous oxygen tension (tcPO2) in healthy subjects while breathing orally and nasally was measured. It was demonstrated that during nasal breathing, 75% of study subjects had a 10% increase in their tcPO2, thus increasing oxygen availability to the lungs and cells.

It is postulated that nasal NO may have local and distal influences, such as improvement in pulmonary function by reducing pulmonary artery resistance (Yu et al., 2019) and participation in host defense mechanisms (García-Ortiz & Serrador, 2018). Available evidence indicates that certain microorganisms are destroyed by a concentration of 100 parts per billion (ppb) of NO (Kartal et al., 2010). By comparison, the concentration of NO in nasal airways is several hundred times higher and can reach up to 30,000 ppb in paranasal sinuses (Maniscalco et al., 2016). This is why nasal NO is considered a component of the first-line defense against respiratory particles and microorganisms (Bath et al., 2021; Kartal et al., 2010; Maniscalco et al., 2016). By extension, this also implicates nasal breathing itself in the first-line defense in preventing respiratory infections (J. O. Lundberg, 2008; Törnberg et al., 2002; Weitzberg & Lundberg, 2002). However, to the best of our knowledge, no clinical trial has directly compared respiratory infection rates between nasal and oral breathers; the existing evidence links nasal breathing to higher airway NO concentrations rather than to infection outcomes.

Separately, some research on NO administered at pharmacological concentrations suggests that NO may protect against coronaviruses, though the evidence spans in vitro and clinical studies with varying levels of support. A review by Lisi et al. (2021) concluded that in vitro studies demonstrated that NO inhibits the replication of both SARS-CoV and SARS-CoV-2, the viruses responsible for the original SARS epidemic and COVID-19. In a complementary in vitro study using airway epithelial cells, Akaberi et al. (2020) found that NO inhibited SARS-CoV-2 replication. Clinically, one study reported successful treatment in COVID-19-infected patients using inhaled NO (Zamanian et al., 2020). However, a systematic review found that while NO improved oxygenation, other clinical outcomes including ventilation duration and mortality did not show improvement (Prakash et al., 2021). Thus, the efficacy against the SARS-CoV viruses appears to be partial and unpredictable, as NO has a complex role in the immunological host responses to viral infections depending on its concentration and the type of pathogen (Lisi et al., 2021). The heterogeneity of study designs, model systems, and outcome measures limits definitive conclusions. On the other hand, the evidence for the antimicrobial impact of NO is more generally robust for a broad range of other pathogens (Bath et al., 2021).

### 3.4. Breathing Practices and Autonomic Regulation

Beyond the body’s endogenous NO production, specific breathing practices integral to many meditative traditions can substantially enhance these protective and regulatory effects. Clinical evidence indicates humming, for example, may increase NO production up to 15 times more than quiet nasal breathing (Weitzberg & Lundberg, 2002), while breath-holding for 30 s after exhalation increases NO concentration and improves nasal obstructions with long-lasting effects (Benedict et al., 2023).

In addition to enhancing NO production, specific breathing practices can directly influence autonomic regulation. Coherent or resonance breathing—characterized by a breathing rate of approximately five to six breaths per minute to maximize HRV—has shown promise as an autonomic regulation technique (Zaccaro et al., 2018).

Similarly, unilateral nasal breathing—a component of yogic pranayama, which encompasses a range of breath-control techniques including alternate nostril breathing (*nadi shodhana*), extended exhalation, and breath retention (Trivedi et al., 2023; Zaccaro et al., 2022)—has been associated with increased parasympathetic tone and HRV, attenuated sympathetic activity, and collectively lower stress levels. Although right nasal breathing has been linked to reduced stress perception and relaxation (Vanutelli et al., 2024), left nostril breathing may improve sleep quality and reduces hyperarousal (Gajbhiye et al., 2022). On the basis of these findings, unilateral nasal breathing has gained clinical recognition and interest, although, while these studies are promising, the conclusions that can be drawn remain limited by small sample sizes, brief intervention periods, and the absence of blinded control conditions (Trivedi et al., 2023; Vanutelli et al., 2024).

Consistent with this, structural abnormalities that interfere with natural nasal airflow and olfactory function, such as nasal septal deviation, may disrupt sleep, reduce quality of life, and increase rates of depression, anxiety, irritability, interpersonal conflicts, and somatization. Symptom improvements have been observed after surgical procedures restoring normal airflow (Alghamdi et al., 2022). Together, this evidence indicates that nasal airflow plays a meaningful role in the balance of the autonomic nervous system through multiple pathways including NO production, vagal stimulation, and direct autonomic regulation, affecting emotional wellbeing, stress resilience, sleep quality, and daily functioning.

### 3.5. Nasal Immune Defense Beyond Nitric Oxide

The nose, more precisely the sinonasal epithelium lining the nasal cavity and sinuses, is filled with bitter taste receptors that not only help with sensory taste and detection of noxious substances but also assist in local immunological responses by detecting the bitter substances created by some bacteria for communicative purposes with each other (Maina et al., 2018; Mao et al., 2023). Nasal mucosal innervation by sympathetic nerve fibers affects local vascular and glandular functions. Nasal gland secretions contain antimicrobial peptides that initiate anti-inflammatory and neurogenic processes using peptides such as histamine and interleukins (Laudien et al., 2011). Secretions absorb inhaled irritants and neutralize microbes or their toxins and potentially facilitate sending informative messages to the central nervous system, thus protecting the respiratory apparatus and enhancing the body’s defense against airborne infections (Cole et al., 1999; Kurtz et al., 2004).

These defense mechanisms are complemented by the nose’s extensive sensory apparatus. The sensory information in the nose is detected by chemo and mechanoreceptors. It is reported that each human possesses approximately ten to twenty million chemoreceptors in their olfactory epithelium (Glezer & Malnic, 2019). Chemoreceptors detect and transduce odors, while mechanoreceptors, which are widespread in the nasal mucosa, detect physical or mechanical sensations like pressure, touch, and airflow. The number and function of both receptor types are influenced by different variables, including age, health, genetics, personal habits (smoking or unhealthy living), or professional environments such as those of miners and firefighters (McClintock et al., 2020; Sharma et al., 2019).

### 3.6. Olfaction and Limbic System Connectivity

Olfaction is a primordial sense and its connection to the brain predates the development of the thalamus needed for relaying and filtering the sensory messages for higher order information processing to the neocortex (Shepherd, 2010). It connects to the amygdala and the hippocampus directly through the olfactory bulb found within the skull right above the nasal cavity (Wilson & Sullivan, 2011). Before the dominance of visual and auditory systems, this chemical sense connected vertebrates (humans included) directly to the environment to detect predators through their scent for safety, food for nourishment, and attract mates for procreation, all for survival.

Under these critical circumstances, proper usage of every millisecond is invaluable and there is no time for the activation of higher-order, intensive cortical processing. The amygdala must react and activate the hypothalamus and sympathetic nervous system for immediate action in the face of threat (fight or flight) or activate reward system circuitry, including the parasympathetic nervous system, in the face of pleasant and joyful experiences (food or mate), all of which occur outside of conscious awareness (Stevenson, 2010). The visual and auditory systems, with their quantitative fine spatial discriminatory properties, are secondary to the sense of olfaction as a qualitative “good or bad” sense (Rolls, 2004).

It is estimated that only about fifty to seventy percent of odors detected by the nose are consciously recognized. Odors not only impact brain function but also emotional reactions. They regulate mood, cognition, thought content, and behavioral responses consciously and unconsciously (Syrjänen et al., 2019).

Although the olfactory nerve (cranial nerve I) is the main route for odor transmission to the brain, the trigeminal nerve (cranial nerve V) also conducts signals from air pollutants, irritants, and pungent odors. These include alcohol, ammonia, capsaicin from chili peppers (Libreros-Jiménez et al., 2023), black pepper, mustard, menthol, horseradish, onion, and garlic (Bandell et al., 2004). Pungent chemical compounds, along with temperature and mechanical changes, activate transient receptor potential channels which are ion channels located on the cell surface of trigeminal neurons that transduce a wide range of environmental stimuli modalities, from chemical to mechanical changes (Venkatachalam & Montell, 2007). Stimulation by these agents and their intensity varies between the olfactory and trigeminal nerves. Some like capsaicin have no measurable impact on olfactory nerves and some others such as mustard and menthol have strong impact on trigeminal nerves and a weak effect on olfactory nerve (Rui et al., 2025).

Depending on the type of substrate, these stimulations will reach different cortical and subcortical regions of the brain and generate a variety of emotional, physiological, or behavioral effects (Soudry et al., 2011). Similarly, some substrates may stimulate the vagus or glossopharyngeal nerves (cranial nerves X and IX, respectively), which innervate the larynx or pharynx. They generate autonomic and reflexive reactions impacting breathing or swallowing with potentially deadly reflexive outcomes such as bronchospasm and cardiorespiratory arrest (Sibilla & Agarwal, 2018). Through its direct link to the limbic structures and their vast connections to other segments of the brain, olfactory stimulation asserts its neuro-psychophysiological impact in extensive parts of the brain (Merrick et al., 2014; Soudry et al., 2011).

### 3.7. Nasal Breathing, Brain Oscillations, and Consciousness

Neurophysiological studies in humans indicate that nasal mechanoreceptor stimulation during slow-paced nasal breathing has been shown to modulate thalamic and cortical functions and enable the alteration of the level of consciousness in humans (Zaccaro et al., 2022). As the relay center of the brain, the thalamus conveys sensory information to the cortex and delivers motor instructions from the cortex to other parts of the brain and body (Cabrera-Álvarez et al., 2023). The thalamus plays a significant role in respiration. It is directly and indirectly connected to the cerebral cortex and limbic system, including the amygdala and hippocampus (Krohn et al., 2023).

Independent of thoracic breathing, animal and human studies provide evidence suggesting a positive impact of nasal breathing on the brain and the autonomic nervous system with calming outcomes (Horii et al., 2013; Jerath et al., 2015). The process is initiated by the nose’s chemo and mechanoreceptors. This modulating effect originates from the olfactory bulb, which processes electrical signals received from nasal receptors along the nasal pathway (Guyenet, 2014). Beyond its direct connection to the amygdala, the olfactory bulb connects extensively to other cortical and subcortical regions involved in odor perception, often bypassing conscious awareness (Nigri et al., 2013).

In an experimental study by (Perl et al., 2019), using human participants, it was found that inhalation during nasal breathing not only activates the brain’s olfactory networks but also enhances cognitive and visuospatial task performance, even when unrelated to olfaction (Perl et al., 2019). Furthermore, studies have observed an increase in delta and theta brain waves over the entire cortex and specifically within the DMN during slow, rhythmic delivery of air to the olfactory epithelium, simulating the inhalation phase of nasal breathing (Piarulli et al., 2018). This influence, associated with a reversal in the flow of information from posteroanterior to anteroposterior, correlates with alterations in consciousness levels (Piarulli et al., 2018). These findings offer tentative neurophysiological underpinnings for what contemplative traditions from Buddhism to Sufism, with their longstanding emphasis on nasal breathing, have long described as deep altered, meditative states of consciousness.

The DMN comprises specific brain regions that are active during passive and resting moments such as daydreaming, ruminating, and goal-directed mental activities involving memory recall, episodic memory, or future imagination, and introspection (Menon, 2023). Although the DMN primarily involves the association cortex and para-limbic regions, it does not include the motor and sensory cortices (Mantini et al., 2011). Various practices such as meditation, breath work, and mindfulness can influence the activity of the DMN (Zagkas et al., 2023).

Electrophysiological evidence in humans from intracranial recordings demonstrates that nasal breathing can systematically synchronize the neuronal electrical activity within the piriform (olfactory) cortex and key components of the limbic system, including amygdala, insula, and hippocampus (Zelano et al., 2016). This synchronization manifests itself in a rhythmic pattern and is further pronounced during nasal inhalation while diminishing in mouth breathing.

Additionally, investigations have uncovered that nasal inhalation enhances memory retrieval and fear discrimination. As cognitive functions advance, behavioral modification ensues (Heck et al., 2019). Behavioral studies further reveal that nasal breathing modulates the functions of the amygdala and hypothalamus in healthy individuals (Zelano et al., 2016). Beyond its intimate connection to the amygdala, nasal breathing influences odor perception mediated by other brain regions and centers (insula, cingula, and olfactory cortex) and impacts our cognitive and emotional landscape to a greater extent, often beyond our conscious awareness (Lübke & Pause, 2015). Another study also found that nasal stimulation (breathing) serves as a significant link between slow breathing and the brain and autonomic nervous system, influencing psychological and behavioral outcomes (Riazi et al., 2024).

### 3.8. Interoception, Emotion Regulation, and Prefrontal Processing

As part of the limbic system, the insula has a direct connection to the amygdala. It integrates peripheral sensory input, including the sense of smell (Craig, 2002, 2009). It is involved in the subjective experience of smell and human emotional reactions to olfactory experiences (Uddin et al., 2017). Through a process called active interoceptive inference, the brain becomes aware of the body’s functional changes to maintain its emotion regulation and homeostasis (Seth & Friston, 2016; Seth et al., 2012).

Applied to nasal breathing, this framework suggests that, for example, during stressful circumstances in which feelings of safety are disturbed by threat, a cascade of events supporting fight and flight is ignited. Heart and breathing rates increase, blood pressure rises, respiration becomes shallow, and a sense of fear and anxiety settles in. As the individual starts breathing intranasally, intentionally, rhythmically, and slowly, the interoceptive signals become more predictable and a sense of calm and safety is thought to return to the person through this feedback mechanism (Paulus & Stein, 2006, 2010), all mediated by distinct parts of insula and its connections to anterior cingulate cortex, amygdala, hippocampus, hypothalamus, and prefrontal cortex (Barrett & Simmons, 2015; Seth et al., 2012). This process is further supported by concurrent parasympathetic activation and nasal NO production.

The anterior cingulate cortex is another limbic region connected to the amygdala and is involved in emotional and cognitive processing of the sense of smell. In general, it regulates emotional reactions, thought modulation, attention, and decision-making (To et al., 2017). The dorsolateral prefrontal cortex also receives information from the olfactory bulb and is involved in executive cognitive functioning, attention, and working memory, as well as evaluating and interpreting olfactory input (Xia et al., 2021).

Odor type, intensity, and other contributing factors intensely influence the spectrum of reactions and behaviors. These may differ from person to person based on individual experiences, familiarity, and other circumstances (Schäfer & Croy, 2024). Secondary to the robust association of olfaction with the previously mentioned brain structures, the nose has a strong impact on the creation of a spectrum of emotional memories and their elicitation (Fokkens, 2021). The evidence reviewed here suggests the putative influence of nasal breathing on cognition, emotions, and behaviors merits further attention.

### 3.9. Traditional Practices: Historical and Cultural Context

In an early twentieth-century text, the Sufi Master, Hazrat Mir Ghotbeddin emphasized several practices involving the nasal passages (Ghani, 2024). These include cleansing the paranasal sinuses with clean, cold water as a meditative technique, as well as specific breathing exercises involving slow, rhythmic inhalation and exhalation through the nostrils, often coordinated with meditative concentration (Angha, 1956). These practices correspond to specific techniques within the broader Tamarkoz tradition (Bahadorani et al., 2021). This traditional approach warrants further scientific investigation.

## 4. Discussion

This review synthesized evidence across developmental, immunological, autonomic, neurophysiological, and contemplative domains to present an integrated account of the role that nasal breathing plays in human health and wellbeing. Several themes emerge from this synthesis (see Table 2).

First, nasal breathing not only serves respiratory functions: it contributes to maxillofacial development and infant–caregiver bonding (Cudziło et al., 2018; Feldman & Eidelman, 2007), provides first-line immune defense through NO production and mucosal barriers (Lee et al., 2017; Maniscalco et al., 2016), modulates autonomic balance through vagal and trigeminal pathways (Trivedi et al., 2023; Zaccaro et al., 2018), entrains limbic oscillations that enhance cognition and emotional processing (Perl et al., 2019; Zelano et al., 2016), and may modulate consciousness through olfactory epithelium stimulation (Piarulli et al., 2018; Zaccaro et al., 2022).

Second, the convergence between traditional contemplative practices and contemporary neuroscientific evidence is striking, suggesting that these traditions may have been empirically tracking real physiological mechanisms long before they could be measured. This review contributes to the literature by integrating evidence across these domains, with the aim of communicating the importance of nasal breathing to a clinical audience. Although individual domains have been reviewed elsewhere, including NO physiology (Lisi et al., 2021), slow breathing psychophysiology (Zaccaro et al., 2018), and respiratory-limbic coupling (Heck et al., 2019), no prior review, to our knowledge, has attempted this breadth of integration.

A distinction that warrants attention concerns the differential roles of nasal inhalation and exhalation, as this has implications for evidence evaluation, protocol specificity, and the development of breathing interventions. The evidence reviewed here suggests that these phases of the respiratory cycle engage partially distinct mechanisms. The neurophysiological effects on limbic oscillations and cognition appear to be driven primarily by nasal inhalation: Zelano et al. (2016) demonstrated that oscillatory power in the piriform cortex, amygdala, and hippocampus peaked during the inspiratory phase and dissipated during oral breathing, while Perl et al. (2019) found that participants spontaneously initiated cognitive tasks during nasal inhalation, with visuospatial performance enhanced during inhalation trials specifically. The olfactory epithelium mechanoreceptor stimulation emphasized by Piarulli et al. (2018) is likewise driven by the pressure of incoming airflow during inhalation. By contrast, the autonomic regulatory effects—particularly parasympathetic enhancement and improved HRV—appear to depend more heavily on the exhalation phase. Slow, prolonged exhalation is the primary driver of respiratory sinus arrhythmia, the mechanism through which breathing modulates vagal tone (Zaccaro et al., 2018). Similarly, humming, which substantially increases nasal NO output (Weitzberg & Lundberg, 2002), is an exhalation-phase activity. Other functions operate across the full respiratory cycle: air conditioning, filtration, and mucosal immune defense are engaged during both inhalation and exhalation, and the developmental effects of nasal breathing during breastfeeding depend on the rhythmic cycling of the complete breath.

More broadly, this need for specificity extends to empirical investigations of traditional nasal practices. As illustration, in an unblinded preliminary pilot study in our laboratory, healthy volunteers who underwent cold saline nasal lavage—modeled on a technique of the Sufi Master Ghotbeddin (Angha, 1956; Ghani, 2024) described above—were observed to have increased alpha wave activity indicative of a relaxed, calm, meditative state that lasted up to an hour. These preliminary findings are noted to motivate future controlled research rather than as evidence for efficacy.

Several limitations of this review should be acknowledged. The search strategy employed iterative, ad hoc keyword combinations rather than a pre-specified Boolean search protocol, and the breadth of our eligibility criteria—though still guided by relevance and thematic fit—afforded substantial discretion in study selection.

Although this approach is appropriate and justifiable for synthesizing wide-ranging evidence across disciplines (Ferrari, 2015; Greenhalgh et al., 2018), it risks limiting reproducibility and introduces the potential that relevant studies were omitted or that the body of evidence appears more consistent than it is. The quality and empirical strength of the included studies vary considerably: some domains are supported by well-established mechanistic evidence (e.g., Zelano et al., 2016) and well-controlled experimental paradigms (e.g., Perl et al., 2019), while others rest on small-sample studies without blinded controls (e.g., Gajbhiye et al., 2022; Trivedi et al., 2023; Vanutelli et al., 2024) or on in vitro findings whose clinical translation remains unproven (e.g., Akaberi et al., 2020; Lisi et al., 2021). Consequently, specific clinical applications remain tentative, and clinicians should be cautious about making strong therapeutic claims. These considerations are summarized in Table 2, which characterizes the evidence base for each domain reviewed.

## 5. Conclusions

The age-old practice of nasal breathing is deeply embedded in human developmental history and contributes to multiple domains of health and wellbeing across the lifespan. Long before the advent of modern science, nasal breathing was an integral part of spiritual and cultural traditions—traditions that are now finding resonance in the contemporary scientific paradigm (e.g., Bahadorani et al., 2021). Beyond its respiratory function, the evidence reviewed here implicates nasal breathing in immune defense, autonomic and emotion regulation, cognitive performance, and attention. Its impact on healthier facial structure development and creation of healthy and secure associative memories and attachment formation is foundational to healthy functioning. Collectively, the foregoing evidence suggests that nasal breathing may represent an underappreciated yet clinically relevant aspect of human physiology, one that may offer a low-cost, accessible complement to existing treatment approaches. Further controlled research is needed to move from suggestive toward robust clinical recommendations, particularly randomized trials examining specific breathing parameters, mechanisms of action, and optimal clinical populations, as well as designs that isolate the contribution of nasal breathing from other components of traditional and contemporary contemplative practices, such as focused attention, postural positioning, and spiritual or cultural context.

## Figures and Tables

**Table 1:** Search terms organized by thematic domain, anchored to ‘nasal breathing’ and/or ‘nasal respiration’.

Thematic Domain	Search Terms
Developmental Foundations	breastfeeding, infant breathing, craniofacial development
Structural & Evolutionary Anatomy	nasal structure evolution, paranasal sinus function, nasal morphology,
Nitric Oxide & Immune Defense	nitric oxide, paranasal sinus, antimicrobial, nasal mucosal immunity
Autonomic Regulation	vagus nerve, parasympathetic, autonomic nervous system, respiratory sinus arrhythmia
Olfaction & Neurophysiology	olfactory, limbic system, amygdala, nasal respiration,
Consciousness & Interoception	consciousness, default mode network, interoception
Breathing Practices	pranayama, Tamarkoz, coherent breathing, unilateral nostril breathing
Psychological & Emotional Outcomes	emotional regulation, anxiety, stress reduction, wellbeing

*Note*. Domain-specific terms were combined with core terms nasal breathing and/or nasal respiration to focus retrieval. Searches were conducted iteratively within PubMed and PMC, supplemented by backward citation searching. Terms were combined flexibly rather than executed as formal Boolean search strings, consistent with the integrative aims of this narrative review. The final synthesis draws on approximately 70 sources.

**Table 2:** Evidence summary (superscript numbers indicate selected supporting sources; see table notes).

Domain	Key Findings	Putative Mechanisms	Design/Population	Evidence Characterization & Limitations
Developmental ^1^	Nasal breathing during breastfeeding contributes to maxillofacial development, ANS maturation, and infant–caregiver bonding	Mechanical forces on facial structures; vagal/trigeminal stimulation; hormone/peptide release promoting bonding	Cross-sectional and longitudinal cohort studies; clinical case reviews; preterm and full-term infants	No RCTs; indirect evidence linking nasal breathing to developmental outcomes; nasal breathing is a distal effect for many of these outcomes
Structural & Evolutionary Adaptations ^2^	Nasal structures (turbinates, nostril shape, vestibular hairs, sinuses) evolved to optimize air conditioning, filtration, and olfaction	Evolutionary selection for thermoregulation, humidification, pathogen defense; climate-driven morphological variation	Comparative morphological analyses; population genetics; cross-population studies	Descriptive and correlational; establishes evolutionary context through abductive inference; does not test health outcomes
Immune Defense: Nitric Oxide ^3^	NO plays a key role in host defense; sinus NO reaches concentrations (up to 30,000 ppb) sufficient for anti-infective activity; both bactericidal and antiviral effects	NO produced by NOS in sinus epithelium; bactericidal at ≥100 ppb; disrupts viral replication	In vitro (bacterial and viral models); cellular and molecular studies	Molecular/cellular evidence well established; limited direct clinical evidence linking sinus-derived NO to immune-related outcomes in humans
Immune Defense: Mucosal & Innate Barriers ^4^	Nasal bitter taste receptors (T2Rs) detect bacterial signaling molecules, triggering local immune responses; mucosal secretions contain antimicrobial peptides	T2R activation releases bactericidal NO and secretions; antimicrobial peptides neutralize microbes; secretions trap irritants	In vitro and ex vivo nasal epithelium studies; human secretion analyses	Cellular-level evidence well established; clinical significance for immune-related outcomes in intact humans remains inferential
Breathing Practices & NO Enhancement ^5^	Humming increases nasal NO up to 15-fold over quiet breathing; breath-holding after exhalation increases NO concentration and improves nasal obstruction	Oscillatory airflow during humming enhances sinus ventilation and NO release; exhalation breath-hold creates back-pressure, increasing sinus NO diffusion	Small-sample experimental studies; healthy and clinical adult populations	Weitzberg & Lundberg is a landmark study but small; mechanisms are plausible but replications with larger, controlled designs are needed
Autonomic Regulation ^6^	Nasal breathing increases parasympathetic tone and HRV; unilateral breathing improves sleep; nasal obstruction associated with mood disturbance	Vagal stimulation via nasal airflow; lateralized autonomic effects; disrupted airflow alters sympatho-vagal balance	Small-sample experimental studies; crossover and within-subjects designs; observational studies; surgical outcomes; healthy and clinical populations	Promising but limited by small samples, absent blinding, short durations; link between structural obstruction and mood draws on clinical review
Olfaction & Emotion ^7^	Olfactory pathways bypass thalamus, connecting directly to limbic structures; enables rapid emotional processing, mood regulation, emotional memory	Direct olfactory bulb–amygdala/hippocampus projections; odor-evoked modulation of emotional memory and autonomic responses; conscious and unconscious processing	Behavioral and neurophysiological studies; healthy adults	Well-established neuroanatomical pathways; behavioral convergence across paradigms and model systems
Neurophysiology & Cognition ^8^	Nasal inhalation entrains limbic oscillations and enhances memory retrieval, fear discrimination, and visuospatial accuracy	Respiratory rhythm entrains neural oscillations via olfactory bulb mechanoreceptors; phase-dependent modulation of cortical excitability enhances stimulus processing	Intracranial and scalp neurophysiological experiments with behavioral tasks; healthy adults and clinical (epilepsy) samples; includes sleep deprivation paradigm	Robust evidence including rare intracranial data; effects diminish during mouth breathing; epilepsy samples may limit generalizability
Consciousness & Interoception ^9^	Slow nasal breathing modulates DMN activity, reverses cortical information flow, and induces non-ordinary consciousness; interoceptive feedback supports emotion regulation	Olfactory epithelium mechanoreceptors influence DMN/limbic regions; effects distinct from vagal stimulation alone; interoceptive inference mediates emotion regulation via insula–ACC–amygdala	Small-sample within-subjects neurophysiological experiments; healthy non-meditators and experienced meditators	Novel HD-EEG evidence; small samples; interoception framework is theoretical, not directly tested
Traditional & Contemplative Practices ^10^	Nasal breathing central to pranayama, Tamarkoz, and other meditative traditions; contemporary scientific evidence is beginning to converge on health and wellbeing benefits	Thought to be due to effects on autonomic regulation, olfactory-epithelial stimulation, and cognitive-affective mechanisms (attentional, interoceptive, and DMN modulation)	Few experimental studies; predominantly historical and religious primary texts	Historical sources provide cultural context; few direct empirical studies of these practices per se; rigorous designs needed for practice-specific mechanisms

*Note.* Selected Supporting Sources: ^**1**^ (Cudziło et al., 2018; Feldman & Eidelman, 2003, 2007; Gipson et al., 2019; Moral et al., 2010; Trabalon & Schaal, 2012). ^**2**^ (Jankowski et al., 2016; Kelly et al., 2020; Roseman & Auerbach, 2015; Zaidi et al., 2017). ^**3**^ (Bath et al., 2021; García-Ortiz & Serrador, 2018; Kartal et al., 2010; Lee et al., 2017; Lisi et al., 2021 (review); J. O. Lundberg, 2008; Maniscalco et al., 2016). ^**4**^ (Cole et al., 1999; Laudien et al., 2011; Maina et al., 2018). ^**5**^ (Benedict et al., 2023 (review); Törnberg et al., 2002; Weitzberg & Lundberg, 2002). ^**6**^ (Alghamdi et al., 2022; Gajbhiye et al., 2022; Trivedi et al., 2023; Vanutelli et al., 2024; Zaccaro et al., 2018 (systematic review)). ^**7**^ (Fokkens, 2021; Lübke & Pause, 2015; Merrick et al., 2014; Schäfer & Croy, 2024; Soudry et al., 2011). ^**8**^ (Heck et al., 2017; Heck et al., 2019 (review); Perl et al., 2019; Riazi et al., 2024; Zelano et al., 2016). ^**9**^ (Barrett & Simmons, 2015; Paulus & Stein, 2006, 2010; Piarulli et al., 2018; Seth et al., 2012; Seth & Friston, 2016; Zaccaro et al., 2022). ^**10**^ (Angha, 1956; Bahadorani et al., 2021; Ghani, 2024; McAllister, 2020; Zaccaro et al., 2018 (systematic review); Zemmelman, 2017). Abbr.: Autonomic Nervous System (ANS); Default Mode Network (DMN); High-Density Electroencephalography (HD-EEG).

## Data Availability

No new data were created or analyzed in this study. This narrative review synthesizes previously published research. All sources cited are available through their respective publications.

## Note

1 The neuromodulatory effects of NO are attributable to neuronal NO produced by nNOS within the central nervous system (CNS) rather than sinus-derived NO, which, given its high reactivity with a half-life of seconds, is unlikely to reach the CNS at neuromodulatory concentrations (Kourosh-Arami et al., 2020). Sinus-derived NO may nonetheless influence CNS function, but diffusely, through its effects on pulmonary, autonomic, and vascular functions.

## Abbreviations

The following abbreviations are used in this manuscript:

NO	Nitric Oxide
NOS	Nitric Oxide Synthase
tcPO2	Transcutaneous oxygen tension
ppb	Parts per billion
HRV	Heart rate variability
DMN	Default mode network
